# Barriers and facilitators to implementing workplace health and wellbeing services in the NHS from the perspective of senior leaders and wellbeing practitioners: a qualitative study

**DOI:** 10.1186/s12889-018-6283-y

**Published:** 2018-12-10

**Authors:** Helen Quirk, Helen Crank, Anouska Carter, Hanna Leahy, Robert J. Copeland

**Affiliations:** 1 Centre for Sport and Exercise Science, Faculty of Health and Wellbeing, Sheffield Hallam University, Collegiate Hall, Collegiate Crescent, Sheffield, S10 2BP UK; 2 Carnegie School of Sport, Leeds Beckett University, Headingley Campus, Leeds, LS6 3QU UK; 3 The National Centre for Sport and Exercise Medicine, Sheffield, UK

**Keywords:** Workplace health promotion, Health and wellbeing, National Health Service, Interviews, Qualitative research

## Abstract

**Background:**

The National Health Service (NHS) seems appropriately placed to be an exemplar employer in providing effective and proactive workplace health and wellbeing services for its staff. However, NHS staff sickness absence costs an estimated £2.4 billion. Evidence suggests staff health and wellbeing services delivered in the NHS can improve health, productivity and sickness absence and yet the adoption of these services remains a challenge, with few examples nationally. This research aimed to explore the perceptions of NHS senior leaders and health and wellbeing practitioners regarding barriers and facilitators to implementing workplace health and wellbeing services for staff in the NHS.

**Methods:**

Semi-structured interviews were conducted with NHS staff, consisting of four senior leaders, four heads of department and three health and wellbeing practitioners in one region of the UK. Interviews were transcribed verbatim and analysed using thematic analysis.

**Results:**

Themes describe the experience of delivering workplace health and wellbeing services in the NHS, and barriers and facilitators to implementation from senior decision makers. Barriers to implementation of services include; a busy and pressurised environment, financial constraints and reluctance to invest in staff health and wellbeing. Barriers to staff engagement were also reported and include difficulty of access to health and wellbeing services and lack of time. Initiating services were facilitated by financial incentives, a supportive organisational structure and culture that takes a preventative, rather than reactive, approach to staff health and wellbeing. Facilitators to implementing health and wellbeing services include a coherent, strategic approach to implementation, effective communication and advertisement, being creative and innovative with resources and conducting a needs analysis and evaluation before, during and after implementation.

**Conclusions:**

Barriers to the successful initiation and implementation of health and wellbeing services in the NHS are numerous and range from front-line logistical issues with implementation to high-level strategic and financial constraints. Adopting a strategic and needs-led approach to implementation and ensuring thorough staff engagement are amongst a number of factors that facilitate implementation and help overcome barriers to initiation of wellbeing programmes in the NHS. There is a need for a culture that supports staff health and wellbeing in the NHS.

## Background

### Workplace health promotion

Individuals on average spend nearly two thirds of their waking hours at work and an estimated 60% of the world’s population is accessible directly or indirectly through the workplace [1]. It is therefore unsurprising that numerous international charters and declarations [2–4] point towards the workplace as an appropriate setting for engendering health and wellbeing (HWB) among its workers [1].

It is also important to recognise that being in work is in itself good for health. Work is important to an individual’s identity, social status, health and economic wellbeing and moreover being out of work has significant and adverse health consequences [5]. That said, a focus on workplace HWB is much needed as recent data suggest a total of 175 million working days (3.3% of total working time) are lost to sickness absence per annum - equating to £14.4 billion in costs to the employer (roughly £495 per employee) [6]. Somewhat ironically, sickness absence rates are higher in the National Health Service (NHS) than other UK employment sectors and so the health of NHS staff as well as that of its patients should be a priority for policy makers [7].

### Workplace health and wellbeing in the NHS

The NHS is one of the world’s largest employers and relies upon a healthy and engaged workforce to deliver its services [8]. In the 2014 Commonwealth Fund report of 11 countries, the NHS was ranked the best healthcare system in the world for its quality of care, efficiency and low cost at the point of service, yet in the same report the UK ranked 10th out of 11 countries on the ‘Healthy Lives’ indicator, the biggest predictor of which is staff wellbeing [9]. Absenteeism in NHS workers has been attributed to low staff wellbeing and in 2015, Public Health England estimated the cost of sickness absence to the NHS at £2.4 billion, demonstrating clear financial incentive to improving NHS staff HWB [10].

As well as staff absence, direct correlations exist between NHS staff HWB, staff productivity and performance, workplace accidents and errors and patient outcomes (e.g., patient experience and patient health outcomes such as MRSA rates) [7, 11, 12]. Data suggest that an NHS Trust in the top 10% for staff HWB is also likely to be in the top 20% for patient satisfaction [11]. This combined with a staff absenteeism rate in the NHS double that of the national average (at a cost of £1153 per person, per annum; [13]) and the challenges of an ageing workforce [14, 15], means there are clear incentives for staff, patients and NHS organisations for implementing effective workplace HWB services in the NHS.

In 2009, the Department of Health requested that the NHS ‘*be an exemplar employer in ensuring the health and wellbeing of its staff…for other organisations to follow*’ [16]. The ‘NHS Five Year Forward View’ [17] and the ‘Commissioning for Quality and Innovation (CQUIN)’ guidance for the NHS [10], continue to underline the importance of the staff HWB agenda and place responsibility on workplaces to offer HWB services and on individual employees to maintain and/or improve their own health [18].

The National Institute for Health and Care Excellence (NICE) has published several evidence-based guidelines for employers on how to improve the health of staff (management of long-term sickness absence, mental wellbeing, obesity, smoking cessation and physical activity in the workplace) [19–24]. Data from controlled and uncontrolled studies has revealed workplace HWB services can maintain good health status among employees over the long-term [25] and deliver significant changes in alcohol consumption, nutrition, sleep, stress, body mass index, depression and perceptions of general health [26–28], including some long-term improvements in body mass, waist circumference, blood pressure and lipid profiles compared to controls [29]. Several studies have also reported positive outcomes of workplace HWB services delivered in the NHS, including long-term changes to physical activity (in and out of work), significantly lower sickness absence, greater job satisfaction and greater organisational commitment [30–34]. Whilst this body of research has demonstrated the potential value of workplace HWB services to the NHS the implementation of these programmes remains a challenge, with few examples nationally.

Understanding the barriers and facilitators to the implementation of workplace HWB services in the NHS could develop our understanding of ‘what works’ and ‘what doesn’t work’ when it comes to staff HWB programmes. Early insight into these barriers and facilitators to HWB in the NHS [7, 35–37] indicates that staff time restraints are a universal concern, with problems relating to shift work, scheduling and work conflicts. The NHS workplace environment has also been cited as a major barrier to engaging in HWB practices, such as canteen opening times, lack of healthy food choices and lack of breaks limiting the healthy eating behaviours. A national audit of how well NHS Trusts in England were implementing the NICE workplace guidance identified; strong organisational values, Board involvement, and having a proactive HWB Board Lead for wellbeing as key elements of successes for the implementation of workplace HWB services [38]. There appears real value in further exploring the barriers and facilitators to adoption of HWB services in the NHS to help overcome some challenges to wider adoption.

Considering the potential value, the current research explores the experience of initiating and delivering workplace HWB services in local NHS Trusts, focussing on the barriers and facilitators to effective implementation. Through a qualitative framework, the research examines the experiences and views of senior leaders and practitioners and explores to what extent the ambitions of the NHS Five Year Forward View and the staff HWB Commissioning for Quality and Innovation (CQUIN) payment framework have been impactful.

The purpose of this study was to explore senior leaders’ and workplace wellbeing practitioners’ perceptions of:

- Barriers to implementing workplace HWB services in the NHS
- Facilitators to implementing workplace HWB services in the NHS
- The ideal implementation of workplace HWB services in the NHS

## Methods

Data were collected by semi-structured interview with senior leaders and workplace wellbeing practitioners across the NHS in one region of the UK. The research was reviewed and approved by Sheffield Hallam University local research ethics committee.

### Recruitment

Senior leaders and workplace wellbeing practitioners were recruited between August and October 2016 using a purposeful sampling procedure. Individuals working in appropriate roles within the NHS were highlighted and contacted directly by the project team through local network contacts. Interested individuals were provided with an information sheet and consent form. A mutually convenient interview time and method (telephone or face-to-face) was arranged. Written consent was received prior to the interview. Verbal consent was confirmed before the interview.

Thirteen people expressed an interest to participate. Written consent was received from 12. The number of participants interviewed was based on the number needed to achieve theoretical data saturation [39]. With each interview conducted, the research team judged whether the data emerging was new and satisfying the research purpose. The researchers deemed no new data to emerge at the eleventh and twelfth interview, at which point recruitment ceased.

### Participants

The 12 participants were recruited from seven NHS Trusts within a single NHS region. The sample consisted of four senior leaders, four heads of department and three workplace wellbeing practitioners (See Table 1). Interview length ranged from 13 to 34 min and the mean duration of interview was 24 min.

**Table 1:** Participant characteristics

Participant	Position/job role	Abbreviation
001	Head of department: Human Resources or Occupational Health	HR
002	Head of department: Human Resources or Occupational Health	HR
003	Senior leader	SL
004	Senior leader	SL
005	Senior leader	SL
006	Workplace wellbeing practitioner	P
007	Head of department: Human Resources or Occupational Health	HR
008	Workplace wellbeing practitioner	P
009	Senior leader	SL
010	Head of department: Human Resources or Occupational Health	HR
011	Workplace wellbeing practitioner	P

#### Data collection

Interviews took place between August and October 2016. Four interviews were conducted face-to-face and eight via telephone. Interviews were recorded using a digital sound recording device. Interviewers (HL (*n* = 3) and AC (*n* = 9)) were female and trained in interview techniques. HL was an experienced researcher with a background in workplace wellbeing in the NHS. AC was a principle researcher with a background in the development, management and delivery of workplace wellbeing programmes.

An interview schedule was employed to ensure consistency across interviews. Questions in the schedule included:

- *Tell me about the current health and wellbeing strategy for staff in your Trust?*
- *What would you ideally like to see in place for your staff in terms of wellbeing support?*
- *What is the main barrier preventing you from implementing that (“ideal” programme)?*

The interview schedule for practitioners was adapted to cover implementation of workplace HWB services and included additional questions such as:

- *What do you provide for staff wellbeing?*
- *What are the challenges in doing that?*
- *What would you ideally like to see in place for your staff in terms of wellbeing support?*

#### Data analysis

Digital recordings of the interviews were transcribed verbatim using an external transcription company. Eleven interviews were transcribed due to a technical failure on one of the interview recordings. Eleven transcribed interviews were analysed by two different members of the research team; HC and HQ. Interview data were analysed using Braun and Clarke’s (2006) six-stage process of thematic analysis. The computer software programme NVivo 11 (QSR International, 2016) was used to facilitate the organisation of codes and themes.

Data analysis began with an inductive approach. Deductive codes relating to specific areas of interest were then looked for in the data. Examples of deductive codes included ‘schemes and funding’ (e.g., CQUIN payment framework), as we were interested to explore the perceived influence of such schemes on the implementation of workplace wellbeing services. HQ led the data analysis, with support from HC and one of the interviewers helped verify the identification and refinement of themes (HL).

In the report of the findings, verbatim quotes with occupation type in parentheses have been used to represent each theme and subtheme.

## Results

Four overarching themes were identified; two representing barriers and two representing facilitators.

**Table:** 

Overarching theme	Sub-theme
1. Barriers to the implementation of HWB services in the NHS	1a. Busy and pressurised environments caused by staff shortages
	1b. Financial barriers to implementation of HWB services
	1c. Perceptions of spending priorities - patients before staff
2. Barriers to staff engagement with HWB services in the NHS	2a. Logistical barriers due to the nature of NHS work
	2b. Dependence on the existence of a receptive audience
3. Facilitators of the implementation of HWB services in the NHS	3a. Government schemes and funding as incentives
	3b. An organisational structure that supports staff HWB
	3c. An organisational culture that supports staff HWB
4. Facilitators of successful delivery of HWB services in the NHS	4a. Coherent, strategic approach to implementation
	4b. Communication and advertisement
	4c. Being creative and innovative with resources
	4d. Needs analysis and evaluation

### Theme 1. Barriers to the implementation of HWB services in the NHS

The first theme captures respondents’ perceptions of the overarching barriers to the implementation of workplace wellbeing services in the NHS. Senior leaders (SL), heads of HR department (HR) and practitioners (P) all referred to the current state of the NHS, and described “*times of austerity*” (001, HR) as having a negative impact on their Trust’s ability to effectively implement staff HWB services. The theme is represented as three main barriers:

1. Busy and pressurised environments caused by staff shortages
2. Financial barriers to implementation of HWB services
3. Perceptions of spending priorities - patients before staff

#### Sub-theme 1a. Busy and pressurised environments caused by staff shortages

Respondents referred to NHS staff shortages and described the negative impact that this had on staff HWB: “*the workplace is under huge pressure and that isn’t going to go away because of the difficulties of attracting and retaining staff*” (005, SL). One HR Lead explained how a workforce that needs HWB services is not necessarily a workforce that will be receptive to such services; “*the demand [for workplace wellbeing services] continues to grow”,* but, *“people are knackered, and that doesn’t always put you in the right frame of mind to want to take advantage of exercise or wellbeing*” (001, HR).

Another perceived consequence of staff shortages was that those people responsible for the implementation of HWB services were also under pressure. This was perceived to have negative consequences on their ability to deliver effective and resourceful HWB services, as explained by one head of HR:

*Everybody who is delivering those services is running to a standstill and we don't necessarily have the time to step back and say actually, could we do this in a better way, could we actually deliver this by doing things differently maybe even free up some resources to do things.*

010, HR

#### Sub-theme 1b. Financial barriers to implementation of HWB services

Respondents referred to financial constraints and how lack of financial resource compromises the ability to invest in HWB services. One senior leader described, “*the worst funding shortage in NHS history…as being the major barrier…we had fairly significant plans contained within the [health and wellbeing] strategy and then our financial situation in the Trust got considerably worse*”(004, SL). Practitioners also identified the financial deficit as being a major barrier, suggesting that having funding, “*breaks down the biggest barriers [to workplace HWB services]*” (006, P) and enables HWB teams to invest in the necessary resources and equipment to deliver a successful workplace HWB service.

#### Sub-theme 1c. Perceptions of spending priorities - patients before staff

Another barrier to NHS workplace HWB services was the perception that the money available in the NHS should be prioritised for patient care rather than staff HWB. Some respondents held the belief that the NHS is traditionally viewed as a service that cares for and invests in services for patients, not its staff. Some respondents (*n* = 4) expressed a concern that compared to a private organisation, the NHS as a public body would be criticised by the media and general public for prioritising staff HWB initiatives over patient care:

*In the private sector, health and wellbeing can be supported, because at the end of the day it’s being paid for out of shareholders’ money; in the NHS I think there is awareness that because we’re a public sector employer we are actually spending taxpayers’ money.*

010, HR

*I think if you’ve got a story out there and the press get a hold of it or, I don’t know, £40,000 was spent on health checks for staff, but actually then the next story is we’ve got people waiting in A&E on trollies, there would be that whole thing of, well, why are they spending money on health checks for staff?*

007, HR

### Theme 2. Barriers to staff engagement with HWB services in the NHS

Respondents identified two main barriers to staff engagement with workplace HWB initiatives:

1. Logistical barriers due to the nature of NHS work
2. Dependence on the existence of a receptive audience

#### Sub-theme 1a. Logistical barriers due to the nature of NHS work

Respondents referred to fundamental characteristics of the NHS work environment that made staff engagement with workplace HWB services difficult. The main logistical barrier was believed to be the time constraints associated with shift work. Long shifts limited time for staff engagement with HWB services and variable shift patterns produced a demand for HWB services 24 hours a day, 7 days a week. One head of HR explained the time barriers associated with staff engagement with exercise classes:

*You’ve got people working different shift patterns...Which is one of the reasons why I think some of our exercise classes are not working, because the people that have said they want them want them at eight o’clock at night or nine o’clock at night or six o’clock in the morning or seven o’clock in the morning when, you know, when they finish their shifts. And I think that’s the problem is we’ve got such a diverse workforce.*

002, HR

Respondents also identified logistical issues that made access to HWB services difficult. In Trusts situated across multiple sites, some staff members did not have direct access to on-site HWB facilities and services. Access to facilities was also impeded by the limited space available on site. One practitioner described the logistical barriers to staff engagement with HWB services:

*With some of the exercise classes, because we are over three different sites that's been met with a little bit of resistance. And although class numbers have been good, I think because people do shifts they, it's not at a reasonable time or because we haven’t been going to the [other] sites, people have taken offence to that. So we're having to work around it.*

008, P

#### Sub-theme 2b. Dependence on the existence of a receptive audience

The perceived barriers to staff engagement in HWB services were not always about logistics of delivery or organisational infrastructure, but related to individual-level motivation in the workforce. Some respondents held the belief that staff members should be held personally responsible for their own health. The common belief was that staff, “*have got to be motivated to do it*” (003, SL) and that generally speaking, they “*don’t have an interest in it*” (008, P). Consequently, the success of HWB services was believed to be dependent on individuals understanding the importance of HWB, taking personal responsibility and being receptive to workplace HWB services. One senior leader explained:

*There are a lot of unhealthy people in the NHS and I think that it comes down to personal responsibility. And I think maybe if they knew what impact it was having on their health they may have taken more of a responsibility.*

009, SL

### Theme 3. Facilitators of the implementation of HWB services in the NHS

This theme captures the three overarching enablers to the implementation of workplace HWB services in the NHS:

1. Government schemes and funding as incentives
2. An organisational structure that supports staff HWB
3. An organisational culture that supports staff HWB

#### Sub-theme 3a. Government schemes and funding as incentives

All respondents were aware of government schemes and funding initiatives such as the Five Year Forward view, commissioning for quality and innovation (CQUIN) and local funding awards. Most respondents were ambivalent about whether government schemes and initiatives were an incentive to initiate change in workplace HWB services in the NHS. Such incentives were considered to have mixed, positive, negative or no impact on the Trust’s HWB agenda. Respondents who spoke positively about government schemes and funding perceived it to be a catalyst for change because they:

1. Raise awareness about the importance of HWB at the Executive Board level
2. Are a powerful incentive
3. Encourage consistency across organisations

Respondents believed that government schemes such as CQUIN raise awareness about the importance of staff HWB and as a result, workplace HWB services are prioritised at the Executive Board level. One HR Lead explained how CQUIN helped to prioritise the HWB agenda at the Executive Board level:

*In October our bombshell hit. And if I’m being honest it totally wiped the health and wellbeing item off the agenda. It wasn’t top of the priority list. It was on mine, but I wouldn’t say it was at, at Board level or, or managers’ level and I think particularly the CQUIN has brought it back to the table, because there’s a penalty now if we don’t achieve what we’ve been asked to achieve.*

002, HR

There was consensus among four practitioners and heads of HR that government schemes were a powerful financial incentive, as explained by one HR Lead:

*In a cash-strapped service it is sadly the reality that you have to have some sort of financial motivation to do it. So, from that point of view both the CQUIN and the Healthy Workplace Initiative that offered us match funding to do things is very helpful.*

010, HR

One senior leader mentioned that government schemes and initiatives make HWB services consistent across Trusts:

*It's enabled the NHS to move forward together adopting similar approaches in certain areas that are covered by the CQUIN which then means that wherever you go in the NHS you're getting a similar sort of approach, so you're getting a bit of reinforcement.*

004, SL

This structured approach was also considered beneficial by a head of HR:

*We’re signed up to a [name of award] Award. So that is looking at five different areas so around substance use and misuse, healthy weight, mental health and wellbeing, protecting health, so things like cancer, domestic abuse, those kind of things and then health and safety. So that award gives us a real structured approach to how we try and take things forward… We achieved bronze last summer and we’ve just submitted our silver. So that gives us a real structured focus...this is obviously much more holistic and in depth assessment rather than just the NHS Health Check.*

002, HR

Respondents believed that the main drawback about government schemes were that they frequently lacked support from adequate funding or resources. This view was shared by senior leaders and heads of HR, who believed that they did not have the resource capacity to succeed;

*I think [CQUIN] helped in the sense of raising awareness, of course it's not been backed by huge resource, so it's one thing to sort of say that it's important but it's another thing to actually back that and support it properly.*

009, SL

*My frustration is still accessing the resource needed to achieve [government scheme/award] and achieve it well, you know. I think that’s my concern is I’m doing things so thinly and at times don’t think we’re doing things particularly well, because we just haven’t got any additional capacity.*

002, HR

Another negative viewpoint was that the introduction of schemes and funding did not change or add to already-established HWB services. Whilst there was an appreciation that the schemes could be useful for putting HWB on the Executive Board’s agenda, in Trusts where a HWB strategy was already in place, schemes and charters such as CQUIN were perceived as having little impact:

*I don’t think it’s particularly changed the mind-set, but I think because we’re doing quite a lot anyway... the CQUIN is about, what are you going to do in the future, as opposed to acknowledging what you have done already-... so there's nothing really that's prompted us [to change].*

001, HR

#### Sub-theme 3b. An organisational structure that supports staff HWB

This subtheme captured the characteristics of the organisational structure believed to facilitate implementation of workplace HWB services in the NHS. All respondents agreed that support was required at all levels of the organisation; from the Executive Board to the front-line HWB practitioners. Three main levels of support within the organisational structure were believed to help HWB implementation:

1. To have a supportive Executive Board
2. To have managerial engagement
3. To have dedicated HWB staff roles with the relevant skills and expertise

Support from the top-down was considered essential for the successful initiation of workplace HWB initiatives in the NHS. Respondents referred to the importance of “*the Board being on board*” (001, HR) with workplace HWB initiatives. At the Executive Board level, HWB had to be considered a priority. Practitioners, HR Leads and senior leaders believed it was particularly beneficial when the senior leader had an existing interest in HWB (personally and/or professionally) and was a role model for HWB behaviours; “*You’ve got to practice what you preach*” (004, SL). However, enthusiasm and intention from high-level individuals was inadequate to initiate change in the implementation of workplace HWB services, especially when Trusts are constrained by other targets and lack supporting funds and resources. One head of HR explained how senior leaders may value staff HWB, but are under constraint from targets in other areas:

*People pay a lot of lip service to [staff HWB], but I think there are a lot of people in senior positions who say, oh yes, yes, we do value it, but actually then don’t truly understand it, and when they then have constraints put on them around targets to meet, I don’t know, certain cancer services for example, that then becomes the priority because that’s what the press pick up and that’s what’s in the headlines.*

007, HR

Support at the managerial level was also considered key to the success of initiating workplace HWB services. There was consensus across respondents that managers need to value workplace HWB and be supportive of their staff attending and engaging with HWB services, so that they can communicate the value of HWB to their staff. This was described by a practitioner and a senior leader:

*I think if [managers] have an input on directing staff to the appropriate service and getting them interested, then it should work more effectively. But if they don't, then there's a breakdown.*

008, P

*The key to embedding [workplace HWB services] and this becoming a central part of what we do, will be the extent to which we can get our line managers, our middle managers to see it as really valuable in terms of those wider issues which they’re responsible for delivering in terms of caring and cared for staff.*

005, SL

The practitioners who had experienced success with initiating workplace HWB strategies described how managers were receptive and, *“really supportive towards it, really welcoming to come to team meetings and to talk to people about it”* (006, P). It was acknowledged that for managers to fulfil this supportive role, training and upskilling of managers would be required to help them understand the importance of HWB and communicate the messages to their staff:

*That’s the biggest thing with this, with health and wellbeing and part of the CQUIN is around that line manager support, their training, their understanding as to why health and wellbeing is so really important, so that they can then cascade that down to their staff.*

002, HR

Having a dedicated HWB lead role in the organisational structure was important for the successful implementation of workforce HWB services. HR staff in particular believed it was important to have a clearly outlined HWB job role with well-defined expectations. This role was said to benefit from being a protected role (i.e., not given other responsibilities) and benefitted from having relevant skills and expertise in the area of HWB. Respondents spoke about how the HWB role has adapted from the traditional occupational health service (OH) and this change has been met with some conflict and job ambiguity. One head of HR described this challenge:

*I’m trying to change the image of the team; that it’s not an occupational health team, it’s a health and wellbeing team and, you know, they hate the fact that I’ve took occupational health out of their job titles. They’re now 'wellness nurses' and that hasn’t gone down well at all.*

002, HR

Respondents were aware of the different approaches taken by HWB and OH, with HWB taking a more preventative approach than the traditional reactive model of OH. There was consensus among practitioners, heads of HR and senior leaders that a transition towards a more preventative approach is required for the success of workplace HWB initiatives, as captured by one head of HR:

*An analogy I’ve used in the past is that we’ve got this bucket of sickness absence in the Trust and the Trust strategy is to try and empty that bucket by bailing it out. Bailing it out is basically, sadly, terminating people with long-term sickness absence or short, recurrent short-term sickness absence, but nobody has stood back and said, hang on a minute, how do we turn the tap off and in my mind turning the tap off is the preventative bit and that’s where the focus needs to lie. It’s no use carrying on, it doesn’t matter how fast you bail it out, if you don’t turn the tap off it’s going to carry on overflowing.*

010, HR

#### Sub-theme 3c. An organisational culture that supports staff HWB

This subtheme captured the notion that taking a preventative approach to workplace HWB requires a change in the whole NHS organisational culture. Change would involve “*trying to change that culture to make people understand the importance of staff health and wellbeing*” (007, HR). One head of HR and a senior leader gave examples from their own Trusts of how environmental changes could contribute to this cultural shift:

*We swapped the cost of chips and the cost of salad in the staff canteens, because we thought it was ridiculous that chips were cheaper than salad, so we persuaded the catering manager to swap the pricing so that chips were more expensive. And also to move all the chocolate and cakes and things away from the tills and put the fruit and so on nearer to the tills.*

010, HR

*We've been completely smoke free now for about four months...people are not allowed to smoke during working hours.. so that's been fairly effective.*

003, SL

### Theme 4. Facilitators of successful delivery of HWB services in the NHS

This theme captures four facilitators to be the overarching facilitators to successful delivery of workplace HWB services in the NHS:

- Coherent, strategic approach to implementation
- Communication and advertisement
- Being creative and innovative with resources
- Needs analysis and evaluation

#### Sub-theme 4a. Coherent, strategic approach to implementation

A coherent, strategic approach to the delivery of workplace HWB services in the NHS was perceived to be the most desirable model. In addition, having an overarching HWB strategy was synonymous with the prioritisation of HWB and the HWB of staff. Interview data, however, provided an inconsistent picture of HWB strategy, with some Trusts; i) having an overarching HWB strategy in place, ii) having a HWB strategy in place but having limited understanding of it, and iii) no HWB strategy in place or unaware of any specific HWB strategy.

The benefit of having a strategy in place included having a common goal, for example: “*knowing what they’re all working towards within a Trust”* (007, HR). However, the majority of practitioners and senior leaders alike perceived scope for improvement in their Trust’s HWB strategy:

*[The HWB strategy] hasn’t really been picked up, so to be perfectly honest with you there isn’t a decent strategy in place actually. It’s meant to be part of another overarching strategy, but it doesn’t link and it’s the kind of thing that’s out of my control.*

007, HR

In Trusts without a clear HWB strategy, respondents described how the approach to implementation tended to be opportunistic interventions delivered individually, for example:

*It would be helpful to have an overall, an overarching strategy, I think that would be helpful. And we've not pulled it all together, there's lots of different initiatives but pulling it all together would be of benefit I think.*

003, SL

*We have developed a range of interventions, but they’ve tended to develop separately and I think one of the things that we need to think about is how we’re offering our staff a coherent approach to health and wellbeing, and in a sense more of a single portal through which they access that.*

005, SL

A common belief was that an all-encompassing system of HWB services would benefit the strategic approach to implementation. Some respondents suggested a whole-systems approach would be beneficial. This all-encompassing system would involve a smooth referral scheme, whereby staff could be referred to any number of different services offered by the Trust or by the local community (e.g., smoking cessation, financial help etc.). This approach was described as working effectively by one of the practitioners interviewed:

*Because we were part of the healthy living service, we were able to directly refer people in to the healthy living service, so we were able to refer people straight into the stop smoking service, the healthy living service …So that made a big difference I think, as a practitioner, having the ability to refer people and give them that support straightaway as well. …So for example people may have said, I need support with weight loss, so you were able to, with their consent straightaway say to them, right, this is healthy living service, you can be referred straight into it. There’s the gym membership I can offer you, and you were able to show people and direct people straight onto that, which I think was really good as well.*

006, P

#### Sub-theme 4b. Communication and advertisement

Effective communication and advertisement of the HWB services on offer within a Trust was another perceived facilitator. Respondents described the best techniques for effective communication as physically going out to the workforce and having presence on the ground with messages. One head of HR described how the Trust had experienced successful communication in the past with a flu vaccination campaign:

*Interventions in our Trust that have worked well - flu is one that as an organisation, apart from last year, we’ve excelled at over the previous four years and a few people have said to me, why did you do so well? Because we went to them...We physically go out there. We know that staff struggle to even come down to the canteen and get a break, so when we’re putting on displays and events, they just physically don’t do it.*

002, HR

Word-of-mouth techniques for communications and advertisement, as opposed to email communication were believed to be a successful method for making staff aware of the HWB services available.

#### Sub-theme 4c. Being creative and innovative with resources

Respondents described HWB budget constraints and referred to the importance of being creative and innovative with what limited resources were available for HWB services. Creative techniques involved utilising external partners and organisations and being efficient with resources. Respondents who had experienced some success with HWB implementation described how they had fostered good relationships with external partners such as workplace wellbeing organisations, local councils, local gyms and businesses. For example, one head of HR said; “*partnership working and relationship building is so crucially important and often when that’s working well, you can pull in on some of that when you need to pull in on it*” (002, HR). The perceived benefits of forming partnerships such as these included having shared resources, for example:

*I also contacted the Physical Activities Coordinator within [the local council], met up with them, looked at what sort of joint work we could do together, …a bit of a partnership with them so they send us resources that they have on offer.*

006, P

Sharing knowledge and expertise with external partners was perceived as a catalyst for change, for example, *“without their [external HWB organisation] direction we probably wouldn’t have implemented it*” (008, P). The benefits of utilising external partners extended to the ability to receive formal training, for example:

*We’ve got a company came in called [name of company], so again they trained, I think it was about 140 odd managers and that’s about, how do you recognise and spot that individual members of staff are getting a bit anxious and a bit stressed. So it’s to try and prevent, prevent the absences.*

001, HR

Being efficient with available resources was mentioned as important in organisations with constraints on HWB budget. Suggestions for how Trusts can be more efficient with resources included employing administration staff, fostering relationships with on-site Estates services and seeking alternative sources of funding. For example, one senior leader explained:

*We're moving into how we can access softer sources of funding, and OK maybe we can't put a programme on that covers 90% of what we wanted to do so let's, so we're going to put on programmes that cover 5%, but it's a start.*

004, SL

#### Sub-theme 4d. Needs analysis and evaluation

Respondents acknowledged the importance of meeting workforce needs with workplace HWB services and considered a needs analysis to be an important facilitator to implementation. One head of HR explained how the needs analysis can inform the HWB strategy:

*One of the first things I did when I came into post was do some baseline data with some staff first just to kind of ascertain, you know, the health behaviours and status of staff and what they wanted and the type of things they think would help them lead a healthier lifestyle. And used that to inform the strategy, looked at our health profiles and compared them with local health profiles to see just exactly where some of our troubled areas are and also just generally find out what staff want.*

002, HR

Three senior leaders mentioned that previous attempts to engage staff in HWB initiatives have struggled to “*reach the people who really need to do it*” (003, SL), suggesting that a needs analysis might help to identify those people and subsequent solutions.

A robust evaluation of HWB services was regarded as important to demonstrate positive outcomes, support funding applications, give credibility to the HWB service and to improve the service for the future. Respondents valued being able to share successes with employees and the Executive Board, because this was believed to help justify the need for the HWB service. One head of HR described how results from HWB service evaluation could be used to promote staff recruitment:

*[Evaluation] is about demonstrating that we do care about our staff. Our strapline is we care and I think it’s important, particularly within NHS where often we struggle to recruit staff, particularly our nurses and doctors, if one of the unique selling points is going to be that we do care about our staff and we can demonstrate that and we get some recognition for doing that then, you know, that’s really quite important.*

P002, HR

## Discussion

The findings are discussed in terms of the barriers to and facilitators of successful implementation of HWB services in the NHS and the recommendations for workplace HWB strategies in the future.

### Barriers to the implementation of HWB services in the NHS

The findings suggest that financial barriers and staff shortages compromise a Trust’s ability to invest in HWB services. The findings depict a self-perpetuating cycle within the NHS where staff shortage results in a stretched and tired workforce in need of effective and resourceful HWB services, but staff shortages, shift patterns and financial deficits decreases accessibility and usage of HWB services. Staff HWB should be considered a resource worth investing in because the greatest asset to the healthcare system is said to be the people who deliver it [8]. In this financially difficult time for the NHS, this may require scrutiny of what makes an effective HWB intervention, reallocation of resources and changes to the ways or patterns of working [40].

The results show that despite financial constraints, HWB services are being implemented in the NHS, but that staff engagement with these services can still be low. This suggests characteristics of the NHS workplace such as shift work, time restraints and multi-sites make the implementation of and engagement with workplace HWB services problematic. Initiatives to overcome time restraints may require Trusts to allocate staff protected time during shifts for HWB engagement. Barriers to staff engagement also involved a perceived lack of motivation or personal responsibility. Methods of engaging NHS staff with HWB services may require a more supportive workplace environment that facilitates staff engagement and encourages staff to feel empowered to make their own decisions about their health behaviours both inside and outside of work [41].

### Facilitators of the implementation of HWB services in the NHS

The findings have demonstrated a number of facilitating factors to implementation and delivery of workplace HWB services in the NHS which may help to overcome some of the barriers. The respondents referred to government schemes and incentives such as CQUIN as potential catalysts for change. Incentives were deemed helpful in bringing staff HWB back to the Executive Board’s agenda, but less helpful if they are not supported with adequate resources. CQUIN underwent an independent evaluation by McDonald and colleagues in 2013 [42]. This evaluation suggested that there were barriers to long-term behaviour change using the CQUIN financial incentives, with the ‘tick box’ nature of the scheme and the ‘all or nothing’ payment rule potentially leading to poor motivation if targets were not met [42]. The success of schemes and initiatives such as CQUIN may depend on there being a supportive culture of HWB within which to embed them effectively.

A key enabler to successful workplace HWB services in the NHS was having an organisational culture and structure that promotes and supports a healthy workforce. It was beneficial to have HWB embedded at all levels of the organisation. For example, Trusts were successful when they had Executive Board involvement in HWB initiatives and when they trained and encouraged managers to support HWB initiatives and cascade this to their staff. This supports the recommendations for management practices outlined in the 2015 NICE guidelines for ‘Workplace health: management practices’ [43]. This document outlines the important role line-managers play in protecting and improving the health of NHS employees and the need for appropriate line manager training is highlighted. Areas such as improving knowledge on the impact staff HWB can have on organisational performance alongside the need for managers to be able to identify and provide additional support to employees who show signs of poor mental or physical health are outlined as important. This is further supported by guidelines for managers on the ‘NHS employers’ website [44]. Research into mental health line-manager training has found it leads to increased knowledge, confidence and ability of line managers to identify and address mental health issues in employees working in both public and private sectors [45].

Respondents also perceived that having an overarching, coherent HWB strategy in place was the primary enabler to successful workplace HWB implementation in the NHS. The success of the HWB strategy is likely to depend on the strategy being communicated to staff across the NHS from senior leader level to front-line staff. Thus, the strategy needs clear communication channels throughout the Trust. Practitioners and HR staff experience difficulty with publicising the HWB service, but find face-to-face advertisement and communication can be effective. In times of financial constraints, Trusts need to be creative and innovative with existing staff and resources. Having administration staff and fostering relationships with on-site Estates services could mean that HWB teams can dedicate their time to delivering the HWB service instead of spending time doing administrative jobs and organising room bookings. Drawing upon the skill and expertise of local external organisations could be a cost-effective use of resources. Such external organisations can facilitate at all levels of implementation from strategy development, provision of services and evaluation. Time and resources should be allocated for needs analysis and thorough evaluation of HWB services. Outcomes should be shared internally and externally and be used to improve the service and enable long-term funding and stability.

Useful resources such as the “NHS Employers” contain information that can help NHS Trusts develop their HWB strategy [44], providing advice and guidance on the ‘how-to’ regarding relevant strategy, engaging with staff, delivering the strategy in addition to evaluation of the strategy.

### A culture of HWB in the NHS

**Fig. 1:**
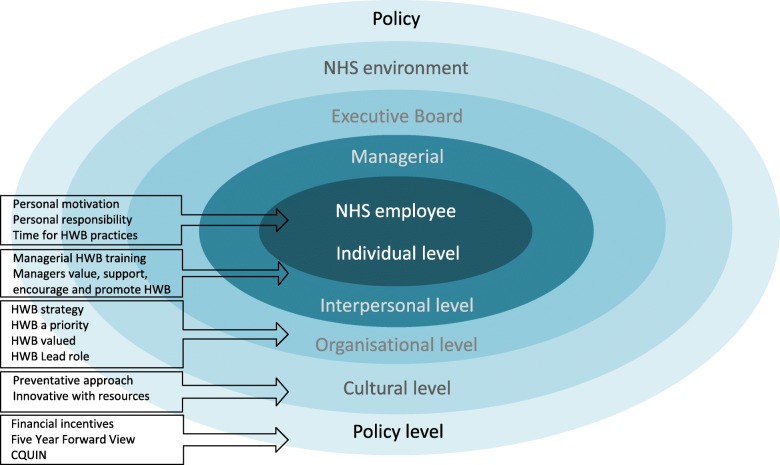
Cultural approach to implementation of staff HWB services in the NHS

Overall, the findings have demonstrated that different levels of influence act upon the successful implementation of workplace HWB services in the NHS. A cultural approach to the design and delivery of HWB services would see staff HWB embedded across the different levels of influence. Fig. 1 shows the findings from this research mapped onto the levels of influence within the NHS and point towards the value of utilising a whole-systems approach to the design and implementation of HWB strategies [41]. The findings are in line with current thinking as the barriers, facilitators and implications discussed are consistent with resources currently available for NHS Trusts (e.g., “NHS Employers” https://www.nhsemployers.org) [44].

### Evaluation

This research provides an in-depth look at the experience of workplace HWB service implementation in the NHS. The findings can be used to extrapolate practical implications for the implementation of HWB services in the future. The methodological rigour supports the credibility of the research, yet findings should be interpreted in light of the following methodological considerations. The results reflect the viewpoint of HWB practitioners and senior leaders in one area of the UK and might not be representative of all NHS Trusts. Further research would benefit from exploring the viewpoint of line managers, as these have been recognised as important influences in staff HWB services. Due to the recruitment methods, the study may have recruited HWB practitioners or senior leaders with a particular interest in workplace HWB, however a broad range of experience and insights were achieved and data saturation was reached.

### Recommendations and implications

Based on the findings from this research the following recommendations are provided:

- The design and delivery of staff HWB services must be set within a culture where HWB is embedded across all levels of the NHS organisation. This culture would benefit from taking a preventative rather than reactive approach to staff HWB.
- Staff HWB strategies should be; informed by insight from a robust employee needs analysis, evidence-based and consider all levels of the organisational system and how levels interact in their design.
- HWB strategy and programmes must be implemented within a robust evaluation and monitoring framework that considers cultural as well as employer-related metrics.
- There is a need for adequate protected or ring-fenced funding for staff HWB services, to enable Trusts to prioritise spending on staff HWB.
- Senior leaders should lead by example when it comes to the engagement in staff HWB.
- Strategies should enable staff to attend HWB services, including flexible service provision to enable shift workers to attend and systems in place to relieve frontline staff to attend services.
- Appropriate line-manager training should be delivered, covering the benefits of staff HWB and the early identification of physical and mental health issues in employees and appropriate action in such occurrences.

### Implications

We also recognise that the findings have implications for the design and implementation of workplace HWB services beyond the NHS. The findings have potential reach into other public service areas such as social care, where a vicious cycle of staff shortage, lack of health promotion and sickness absence could exist. In addressing these issues, it has been suggested that HWB services are cognisant of the physical and mental challenges that occur across the life course of employees [14]. As recommended by other researchers, the findings demonstrate the importance of moving away from this vicious cycle and ‘activating a virtuous cycle through investment in health promotion’ [15] (page 10).

## Conclusions

There is increasing need for improving the mental and physical health of healthcare professionals and NHS staff HWB is a priority. From interviews with senior leaders and HWB practitioners in the NHS, we conclude that there are a number of barriers to the successful implementation of HWB services. The barriers range from front-line logistical issues with implementation to high-level strategic and financial barriers. However, these findings have also demonstrated a number of facilitating factors which may help to overcome some of the barriers. The findings suggest there is a need for a culture that supports staff HWB in the NHS.

## Abbreviations

CQUIN: Commissioning for Quality and Innovation
HR: Head of department (Human Resources or Occupational Health)
HWB: Health and wellbeing
NHS: National Health Service
P: Practitioner (workplace wellbeing)
SL: Senior leader
